# Current Biomedical Use of Copper Chelation Therapy

**DOI:** 10.3390/ijms21031069

**Published:** 2020-02-06

**Authors:** Silvia Baldari, Giuliana Di Rocco, Gabriele Toietta

**Affiliations:** 1 Department of Research, Advanced Diagnostic, and Technological Innovation, IRCCS Regina Elena National Cancer Institute, via E. Chianesi 53, 00144 Rome, Italy; silvia.baldari@ifo.gov.it (S.B.); giuliana.dirocco@ifo.gov.it (G.D.R.); 2 Department of Medical Surgical Sciences and Biotechnologies, University of Rome “La Sapienza”, C.so della Repubblica 79, 04100 Latina, Italy

**Keywords:** copper, chelation therapy, therapeutic chelation, metal homeostasis, cancer, metalloproteins

## Abstract

Copper is an essential microelement that plays an important role in a wide variety of biological processes. Copper concentration has to be finely regulated, as any imbalance in its homeostasis can induce abnormalities. In particular, excess copper plays an important role in the etiopathogenesis of the genetic disease Wilson’s syndrome, in neurological and neurodegenerative pathologies such as Alzheimer’s and Parkinson’s diseases, in idiopathic pulmonary fibrosis, in diabetes, and in several forms of cancer. Copper chelating agents are among the most promising tools to keep copper concentration at physiological levels. In this review, we focus on the most relevant compounds experimentally and clinically evaluated for their ability to counteract copper homeostasis deregulation. In particular, we provide a general overview of the main disorders characterized by a pathological increase in copper levels, summarizing the principal copper chelating therapies adopted in clinical trials.

## 1. Introduction

Copper is an essential trace element involved in a plethora of biological processes in living cells. Analysis of human proteome identified 54 copper-binding proteins—of which, 12 are copper transporters, approximately half are enzymes and one (Antioxidant 1 Copper Chaperone, ATOX1) is a transcription factor [1]. Copper-binding proteins include cytochrome oxidase, copper-zinc-superoxide dismutase, lysyl oxidase, tyrosinase, and dopamine-beta-monooxygenase, which are involved in pivotal biological processes like mitochondrial respiration, antioxidant defense, extracellular matrix cross-linking, pigmentation and neurotransmitter biosynthesis, respectively [2,3]. For an accurate list of copper-requiring enzymes, with particular emphasis on enzymes involved in genetic disorders of copper homeostasis, refer to Horn et al. [4]. The majority of copper in the body is located in organs with high metabolic activity, such as liver, kidneys, heart and brain; approximately 5% of total copper is in the serum—of which, up to 95% is bound to ceruloplasmin (Cp). Unbound copper behaves as a potent oxidant, catalyzing the formation of highly reactive hydroxyl radicals leading to DNA, protein and lipid damage [5]. Therefore, cellular copper concentration needs to be finely regulated by complex homeostatic mechanisms of absorption, excretion and bioavailability [6]. Upon absorption in the gastrointestinal tract, copper reaches the blood, where it is mostly bound to Cp. Copper transporter 1 (*CTR1*, *SLC31A1*), located on the cell membrane, is the main copper import protein; within the cell various metallochaperones receive and deliver copper to specific locations. ATPase copper-transporting alpha (*ATP7A*) and ATPase copper-transporting beta (*ATP7B*) are key players in copper homeostasis being required for copper delivery to the secretory pathway and for efflux of excess copper from the cell. Deregulation of this delicate balance that maintains copper homeostasis has been associated with the pathogenesis of several diseases [7,8]. Consequently, a continuously growing number of in vitro and in vivo studies suggest that copper-involving mechanisms may represent a potential therapeutic target for different pathologies.

## 2. Clinical Application of Copper Chelation Therapy

A chelator is a chemical compound able to selectively bind, due to its structure, a particular atom/ion, with the formation of a stable complex ring-like structure. Metal chelating agents are used as nutritional supplements, for designing radiopharmaceuticals, as additives for cleaning chemicals, cosmetics, plastics, fertilizers, growth supplements in aquaculture, and to remove toxic metals from soil and in the body (chelation therapy) [9]. For a detailed biochemical description of several copper chelating agents, the reader is directed to a previously published review [10]. Copper overload toxicity as well as clinically significant copper deficiency are rare and mostly associated with genetic defects of copper transport such as Wilson’s disease (copper overload) and Menkes disease (copper deficiency). On the other hand, copper is an essential catalytic cofactor in redox biochemistry; consequently, copper dyshomeostasis leading to its unpaired distribution has been linked with several disorders including diabetes, neurological disorders and cancer [11]. Different chelating drugs have been shown to modulate copper levels by different mechanisms; in particular, penicillamine, trientine, and dimercaptosuccinic acid form complexes which are excreted in the urine, while tetrathiomolybdate promotes copper biliary excretion (Table 1). In addition, administration of zinc salts has been suggested as maintenance treatment for Wilson’s disease; zinc interferes with the gastrointestinal copper uptake by inducing metallothionein, which chelates copper, preventing absorption and allowing for its excretion in the feces. The use of copper chelating drugs such as trientine in Wilson’s disease and in cancer patients has been considered safe [12,13]; nonetheless, the specific risk–benefit ratio for each therapeutic indication should be carefully evaluated by additional randomized clinical trials.

The aim of the present review is to provide a global overview on the main different chelation therapy approaches which have been evaluated for the treatment of the diseases in which copper imbalance has a key role in the onset of the pathology, including genetic diseases of copper metabolism such as Wilson’s diseases [8], neurodegenerative diseases such as Alzheimer’s and Parkinson’s diseases [14], idiopathic pulmonary fibrosis [15], diabetes [16], and different forms of cancer [17].

### 2.1. Wilson’s Disease

Wilson’s disease [18], Menkes disease [19] and occipital horn syndrome [20] are human genetic disorders associated with the deregulation of copper-transporting ATPases. Menkes disease and occipital horn syndrome are due to mutations in the *ATP7A* gene, resulting in reduced levels of serum copper and ceruloplasmin [21,22]. The current treatment for Menkes disease is mainly based on parenteral administration of copper-histidine [23]. In contrast, Wilson’s disease is an autosomal recessive disease caused by mutations in both copies of the *ATP7B* gene [18,24] leading to excess copper in the body and characterized by a series of clinical manifestations which include liver failure, tremors and other neurological symptoms [25]. Therefore, to manage increased copper levels, Wilson’s disease patients have been treated with different chelating agents, including D-penicillamine, trientine hydrochloride and tetrathiomolybdate [26,27] (Table 2). The goal of copper chelating therapy for Wilson’s disease is to remove copper accumulated in tissues (de-coppering phase) and to prevent re-accumulation (maintenance phase). Introduced in 1956, D-penicillamine (DPA) [28], a dimethylated cysteine, mobilizes tissue copper stores and promotes efficient excretion of excess copper into urine, but this amelioration of copper balance is not followed by improvements in the neurological symptoms. Instead, DPA treatment may be responsible for worsening patients’ neurological symptoms, due to a putative increase in brain copper level [29]. Furthermore, the use of DPA has been limited by hematologic and renal toxicities [30]. Therefore, DPA was replaced by alternative anti-copper agents such as zinc salt, introduced in 1960 [31] and trientine in 1980 [32]. Zinc salts decrease intestinal dietary copper absorption by inducing the synthesis of intestinal copper chelating peptide metallothionein. Copper is therefore sequestered within the enterocytes and ultimately excreted into feces [33]. Zinc has been added in 1997 by US Food and Drug Administration (FDA) to the list of Wilson’s treatments as maintenance drug [34]. Dimercaptosuccinic acid (DMSA), an antidote to heavy metal poisoning, and DMSA analogues have been extensively used for Wilson’s disease therapy in China because of local availability and affordability [35]. The reported toxic side effects are reduced compared to that of penicillamine [36]; one of the major limitations of DMSA is associated with its inability to cross the cell membrane.

Triethylenetetramine (TETA), also known as trientine, was specifically introduced for the treatment of Wilson’s patients showing DPA intolerance [32]. Trientine has improved safety profile but lower cupreuremic effect compared to DPA. An additional copper chelating agent is ammonium tetrathiomolybdate (TM), which is also able to significantly reduce copper absorption when administered with food [37]. Preclinical studies performed with TM have led to FDA approval for a clinical trial for the treatment of Wilson’s neurological disorders [37,38,39]. In a comparative clinical trial, a clear reduction of the number of patients with neurodegenerative disease in the group treated with TM was determined with respect to the TETA treated group [40]. Despite the potential efficiency and limited toxicity, the clinical use of TM is limited by instability of the ammonium formulation [4] and to low compliance due to frequency of dosing (6 times/day). For these limitations, a derivative of TM, the bis-choline-tetrathiomolybdate, has been recently introduced and a new multicenter phase II study has been performed, demonstrating the efficiency of the drug with no cases of paradoxical drug-related neurological worsening [41]. Moreover, a phase III study comparing bis-choline TM with other copper chelating compounds has been started in 2018 [42]. In recent years, other compounds have been tested in animal models. Among them, DMP-1001 {methyl 4-[7-hydroxy-10,13-dimethyl-3-({4-[(pyridin-2-ylmethyl)amino]butyl}amino)hexadecahydro-1H-cyclopenta[a]phenanthren-17-yl]pentanoate} [43]; methanobactin [44], trientine delivered through liposomes [45] and curcumin [46]. These drugs, however, need further studies both in vitro and in vivo as they have not been used in clinical trials so far. An updated overview on the currently approved treatments for Wilson’s clinical manifestation is reported in several recently published reviews [27,47,48] focused on the efficiency, the side efaafects and possible combination therapies.

### 2.2. Neurological Diseases

#### 2.2.1. Alzheimer’s Disease

Alzheimer’s disease (AD) is the most common form of dementia, characterized by progressive memory loss, language difficulties, disorientation along with recognizable pathological markers including senile plaques and neurofibrillary tangles [49]. From a molecular point of view, AD is characterized by extracellular deposits of β-amyloid protein accumulated in the brain, ultimately leading to neuronal loss [50]. Condensation of β-amyloid in plaques is linked to high concentrations of Cu(II) and Zn(II) in the neocortical tissue, therefore suggesting a role of metal imbalance in the onset of AD [51,52,53]. Moreover, β-amyloid binds and reduces Cu(II) to Cu(I), inducing electron transfer to molecular oxygen with the formation of H_2_O_2_, leading to apoptotic cell death [51]. Copper levels in cerebrospinal fluid of AD patients are 2.2 fold higher than in controls; moreover, increased levels of ceruloplasmin in the brain and in cerebrospinal fluid have been also observed [54]. On the other hand, other studies reveal a significant reduction of copper in hippocampus and amygdala areas, suggesting that abnormal copper compartmentalization in different tissues and organs may be associated with AD [55]. This discrepancy is, at least in part, due to an increase in the free pool of copper with a corresponding reduction in protein-bound copper [56,57]. Interestingly, post-mortem analysis performed on a transgenic mouse model of AD demonstrated that metal chelating agents can attenuate β-amyloid protein excess [58,59]. Clinical trials on AD patients using D-penicillamine [60] and the ionophore PBT2 [61] have been performed (Table 2). Nonetheless, whether copper chelating agents or metal protein attenuating compounds [62] may represent a potential therapeutic solution in AD patients is still a matter for debate [63,64].

#### 2.2.2. Parkinson’s Disease

Parkinson’s disease (PD) is among the most common neurodegenerative disorders, affecting approximately 2–3% of the population over 65 years. The principal hallmark of PD is represented by the typical dopamine-producing neuronal loss in the substantia nigra, accompanied by α-synuclein aggregates usually termed as Lewy bodies, leading to the characteristic symptoms of bradykinesia, muscular rigidity, tremors and other non-motor symptoms. Copper binding to the α-synuclein protein is an important event in the development of PD, triggering protein fibrillation and increased oxidative stress [65,66]. Moreover, the binding of copper to ceruloplasmin is reduced in PD patients, leading to an increase in the levels of free copper, associated with oxidative stress and neurodegeneration [67]. Therefore, copper homeostasis alteration plays a role in PD [68]; however, it remains controversial whether this is the primary cause or a secondary consequence of the disease. Moreover, no consensus has been reached on the possibility to modulate copper content to alleviate PD manifestation since some studies suggest that copper level should be lowered while other studies show the opposite. Several approaches have been tested in the direction of increasing brain copper levels, both regulating copper transporters and administrating copper compounds [69], or “attenuating” copper dysregulation by chelation therapy, recently reviewed by Tosato et al. [70]. In addition to copper, other metals are deregulated in PD, including Fe, Zn and Mn. Accordingly, a wide range of compounds have been proposed for PD therapy [70], including iron chelators [71]. For instance, deferiprone has been widely used for the treatment of systemic iron-related diseases and for neurological pathologies, including PD [72], due to its low molecular weight and ability to cross the blood–brain barrier. Different studies demonstrated deferiprone’s ability to chelate not only iron but also copper, aluminum and zinc [73], reducing their free radical catalytic activity [74]. Moreover, multifunctional iron/copper chelating agents have been evaluated even if their clinical translation has not yet progressed [75].

### 2.3. Idiopathic Pulmonary Fibrosis

Idiopathic pulmonary fibrosis (IPF) is a form of chronic lung disease, usually affecting people between the ages of 50 and 80 years, in which fibrosis progressively build up in the lungs, leading to impairment of lung functions [76]. The wide heterogeneity of clinical manifestations and symptoms leads to a high variability in therapy course and response. The exact mechanism of IPF pathogenesis has not been clarified yet [77]; different biological and molecular factors may be involved including lysyl oxidases, a group of copper-dependent enzymes involved in covalent cross-linking of type I collagen [78]. In particular, LOXL2 may represent a potential therapeutic target, being pro-fibrotic and highly expressed in IPF lung biopsies [79,80]. A study, performed in 2003 [81], demonstrated that administration of TM induced a reduction in serum ceruloplasmin leading to a corresponding reduction of lung fibrosis in a mouse model of bleomycin-induced IPF, paving the way for a clinical trial on IPF patients unresponsive to other therapies (NCT00189176) (Table 2). It has been proved that TM exerts its beneficial effect on IPF by reducing collagen-I expression and accumulation, acting on the expression of the copper-dependent lysyl oxidases [82].

### 2.4. Diabetes Mellitus

Diabetes mellitus (DM) is a group of heterogeneous metabolic diseases mainly characterized by a hyperglycemic condition, with a defect in insulin secretion or action. Three are three main types of diabetes: type I, type II and gestational diabetes [83]. DM patients have higher levels of copper in plasma or serum compared to healthy individuals [84,85]. The development and progression of DM have been associated with an increase in oxidative stress [86] and with imbalance of several metals [87], including copper. Transition between Cu(I) and Cu(II) leads to the production of reactive oxygen species (ROS) and to consequent peroxidation of lipids, DNA damage leading to cell death. Therefore, copper homeostasis maintenance using copper chelators may represent a strategy for diabetes treatment [16]. A series of pre-clinical and clinical studies demonstrated the potential of trientine in reducing some of the clinical and pathological consequences of diabetes, such as heart failure [88]. Another putative therapeutic compound with chelating abilities is metformin, a first-line drug for treatment of type II diabetes which reduces diabetes-related vascular risk. Metformin binds a series of different transitional metals, having a higher affinity for copper [89]. In particular, the interaction of metformin with mitochondrial copper, resulting in the alteration of cellular energy metabolism via inhibition of mitochondrial respiratory chain complex 1, has been proposed as a putative mechanism of action of the drug [90]. Treatment with TM has been described to promote a significant reduction of insulin resistance in the mouse model of type II diabetes C57BL/KsJ-db/db [91]. Two clinical trials employing copper chelating agents for DM have been described, both evaluating trientine treatment effects on macular edema after cataract surgery and diabetic retinopathy: the first (NCT01295073) has been withdrawn and the second (NCT01213888) terminated, with only a few patients enrolled (Table 2). Additional studies are needed to provide definitive conclusions on the utility of copper chelation therapy for DM.

### 2.5. Cancer

Increased copper content has been determined in serum [92] and tissue samples [93] from patients with different types of cancer, including laryngeal squamous cell carcinoma [94], non-Hodgkin’s lymphoma [95], multiple myeloma [96], chronic lymphocytic leukemia [97], hepatocellular carcinoma [98], gynecological carcinoma [99], colorectal [100], lung [101], primary brain [102], and breast [103] cancers. Serum copper levels return to normal upon successful tumor surgical removal or on remission. In addition, gene expression analysis revealed multiple alterations in a variety of copper-binding or copper-sensitive proteins in colorectal [104] and breast cancers [105], suggesting that deregulation of copper homeostasis might contribute to cancer pathogenesis, development and metastasis. Collectively these indications provide support for copper chelation [106,107,108] and inhibition of copper -transporting ATPases [109] as potential strategies for cancer therapy.

As a matter of fact, copper chelating agents used to treat Wilson’s disease such as trientine, penicillamine, and tetrathiomolybdate (both ammonium tetrathiomolybdate, TM, and choline tetrathiomolybdate, ATN-224), revealed chemotherapeutic properties in experimental preclinical cancer models (Table 3) leading to several clinical trials (Table 4). These trials have proved that copper chelation therapy is generally well tolerated, for the reason that copper chelation agents act selectively on cancer cells, which have increased copper content, exerting little toxicity to normal cells [110,111].

#### 2.5.1. Copper Chelation and Tumor Angiogenesis

**Figure 1:**
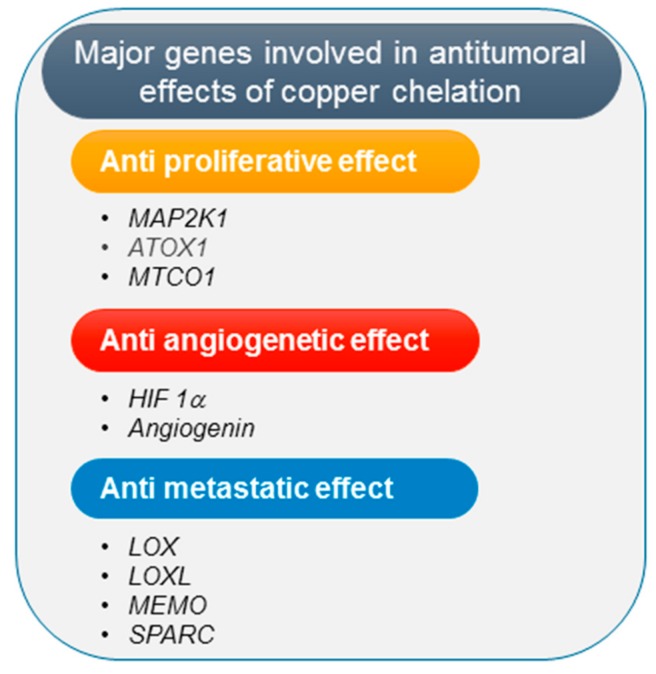
Schematic representation of the main genes involved in the antitumoral effects of copper chelation therapy [1].

Neoangiogenesis is essential to support cancer cells growth and tumor metastasis. The mechanism of cancer inhibition by copper chelating agents is commonly attributed to their inhibitory effect on tumor angiogenesis [106]. In fact, copper stimulates proliferation and migration of endothelial cells [112] and affects mobilization of bone marrow-derived endothelial progenitor cells which promote angiogenesis [113]. Significantly, copper can either directly or indirectly influence different proangiogenetic pathways. In particular, copper can bind angiogenin promoting its biological activity to stimulate formation of blood vessels [114]; moreover, copper is required for the binding of hypoxia-inducible factor (HIF-1) to the hypoxia-response elements, thus modulating expression of some key proangiogenic factors [115], such as vascular endothelial growth factor (VEGF), fibroblast growth factor (FGF), interleukin (IL)-1α and IL-8 (Figure 1).

#### 2.5.2. Copper Chelation and Inhibition of Tumor Proliferation

As an essential catalytic cofactor for proteins, copper is implicated in fundamental biological functions including cellular energy metabolism, growth and development [116]. In particular, among the identified copper-binding proteins [1], the transcriptional factor ATOX1 promotes the expression of the proliferation protein cyclin D1 [117]; accordingly, inhibition of the copper binding protein ATOX1 without depleting the extracellular copper has a critical effect on cancer cell proliferation [118]. Moreover, copper modulates oxidative phosphorylation via cytochrome *c* oxidase activity, affecting cellular growth [119]. Interestingly, it has been demonstrated that copper binding to the mitogen-activated protein kinase kinase 1 (MAP2K1) promotes the activation of the mitogen-activated kinase (MAPK) pathway, which has a prominent role in promoting tumor growth [120]. Accordingly, *BRAF*^V600E^ cancers, which are characterized by increased MAPK pathway activation, have been observed to be sensitive to copper chelation therapy approaches. Preclinical results have been obtained in different tumors, including melanoma [121], lung adenocarcinoma [120], colon carcinoma [122] and papillary thyroid cancer [123], suggesting that copper-chelation therapy could represent a therapeutic option to treat cancers containing the *BRAF*^V600E^ mutation. On these premises, a clinical trial (NCT02068079) to test a copper chelating therapy using TETA in combination with a BRAF inhibitor (Vemurafenib) on *BRAF^V600E^*, late stage melanoma patients has been proposed, but it has been subsequently withdrawn.

#### 2.5.3. Copper Chelation and Tumor Spread

Elevated serum copper levels have been detected in breast cancer patients with distant metastasis [124], suggesting that copper may support the migration/invasion process and increase the metastatic potential of cancer cells. Differential copper levels modulate the activity of the copper-binding enzymes Lysyl Oxidase (*LOX*) and Lysyl Oxidase-Like (*LOXL*) which are involved in the crosslinking of collagen and elastin in the extracellular matrix and whose deregulation has been associated with metastatic progression [125]. In particular, these enzymes are involved in tumor microenvironment remodeling, creating a scaffold for tumor cells as they spread. Moreover, copper binds and activates the Mediator of Cell Motility protein (*MEMO*) which facilitates the migratory capacity of breast cancer cells, thus facilitating metastasis [126]. In addition, copper has also a role in the process of epithelial mesenchymal transition (EMT), in which cancer cells acquire mobility and invasive properties via the HIF1-α-Snail/Twist signaling pathway [127]. Another copper-dependent protein involved in promoting tumor metastasis and invasion is the Secreted Protein Acidic and Rich in Cysteine (*SPARC*) [128,129] (Figure 1). Taken together, these data indicate that copper depletion, acting on the molecular pathways involved in EMT, migration, formation of the tumor microenvironment and pre-metastatic niche, may represent a therapeutic strategy in the treatment of metastatic cancer. Therefore, several clinical trials using copper chelation therapy for metastatic cancers such as colon, breast, lung, prostate cancers and melanoma, have been performed, providing evidence of reduced progression of micrometastases to macroscopic nodules [130,131].

#### 2.5.4. Copper Chelation Combination Therapy Regimens

Several studies suggest that combination therapy strategies for solid tumors based on copper chelation therapy might result in a more effective multimodal approach. In the following section, we review some of the strategies which have been explored.

##### Copper Chelation and Chemotherapy

Chemotherapy drugs are widely used against solid cancers, but although many cancer cells are initially sensitive to chemotherapy, they may develop resistance over time. One of the mechanisms responsible for drug resistance is decreased cellular drug accumulation. Patients not responding to platinum-based chemotherapy have increased serum copper content up to 160% compared to responding patients [132], suggesting a link between maintenance of copper homeostasis and drug resistance. Copper transport proteins play a role in cisplatin, the most used platinum based chemotherapeutic drug [133,134]. Cellular copper homeostasis is accurately regulated by the *CTR1*, responsible for specific copper cellular uptake into cells, and the copper-transporting P-type ATPases (*ATP7A* and *ATP7B*), which are mainly responsible for supplying copper to cuproenzymes and for the removal of excess copper out of the cell. Albeit highly selective, copper transport proteins may also facilitate platinum-drugs cellular influx, accumulation and efflux. As a matter of fact, *CTR1* can also transport platinum drugs into the cell and its expression has been associated with cisplatin sensitivity [135]. Conversely, *ATP7A* and *ATP7B* may promote cisplatin cellular efflux, reducing drug cellular accumulation and leading to reduced efficacy; accordingly, increased expression of *ATP7A* and *ATP7B* correlates with platinum drug resistance [133]. Importantly, expression and activity of *CTR1*, *ATP7A* and *ATP7B* are modulated by intracellular Cu levels. Therefore, copper chelation therapy, reducing cellular copper content and, in turn, increasing *CRT1* and reducing *ATP7A* levels, enhances cellular accumulation and efficacy of chemotherapy drugs [136]. Therefore, different clinical trials have been performed to evaluate copper chelation therapy as a tool to overcome platinum-based drug resistance in cancer patients [137,138,139] (Table 4). In addition, selenium compounds, used both as cytotoxic agents and as adjuvants in chemotherapy [140], exhibit the ability to chelate copper [141]. Another promising class of metal complexes suitable for anticancer therapy is represented by Cu(II) chelate complexes [142]. Although the precise mechanisms of action are yet unclear, there is evidence that copper chelate complexes may act as proteasome inhibitors, superoxide dismutase mimetics, DNA intercalating agents, apoptosis inducers and by promoting ROS production [143]. Clinical translation using this class of compounds is still limited [144,145].

##### Copper Chelation and Radiotherapy

Increased efficacy of radiotherapy against primary tumors with reduced side effects can be achieved when combined with antiangiogenic agents [146]. Along these lines, an additive effect of radiotherapy and copper chelation therapy has been observed in a Lewis lung high metastatic carcinoma mouse tumor model [147].

##### Copper Chelation and Immunotherapy

Immunotherapy treatments have been designed to modulate patient’s own immune system to fight against cancer. There are several immunotherapy strategies, including the use of monoclonal antibodies, immune cell activators, immune checkpoint inhibitors and oncolytic viral vectors. In the following subsections we review the main copper chelation and immunotherapy combination strategies.

##### Copper Chelation and Monoclonal Antibodies Immunotherapy

The monoclonal antibody Cetuximab, which binds specifically to the epidermal growth factor receptor (EGFR) thereby blocking transmission the relative proliferative signaling pathways, is an example of an immunotherapeutic agent. Combination of TM and Cetuximab has been evaluated in a murine model of head and neck squamous cell carcinoma but no statistically significant differences were observed between single and combined treatments [148]. Therefore, further investigations are needed to determine the clinical significance of combining copper chelation and monoclonal antibodies-mediated immunotherapy.

##### Copper Chelation and Immune Activation

Copper chelation has been proposed in conjunction with immune activation for cancer immunotherapy. In particular, Zhou et al. recently developed a copper chelator used to prepare nanoparticles suitable for loading and delivery to the tumor the Toll-like receptor agonist R848, in order to stimulate antitumor immunity by dendritic cells activation. This strategy of nanoparticle-based copper chelation and immune stimulation effectively inhibits breast tumor growth and metastasis in experimental models both in vitro and in vivo [149].

##### Copper Chelation and Immune Checkpoint Inhibitors

An important strategy for cancer immunotherapy targets the interactions between the immune checkpoints programmed cell death protein 1 (PD-1) and the programmed cell death ligand 1 (PD-L1) using specific antibodies. A positive correlation between the copper transport protein CTR1 and PD-L1 expression has been observed in neuroblastoma and glioblastoma tumor cells. Interestingly, copper chelation reduces PD-L1 expression, promoting a significant increase in tumor-infiltrating lymphocytes in a syngeneic mouse model of neuroblastoma [150]. Therefore, copper chelation therapy may promote the efficacy of PD-1/PD-L1 based immunotherapy.

##### Copper Chelation and Oncolytic Virotherapy

Oncolytic vectors selectively replicate and promote lysis of cancer cells triggering the patient’s immune system against tumor antigens. Changes in the tumor microenvironment in response to induced oncolysis may limit the efficacy of oncolytic virotherapy. Therefore, is has been hypothesized that combination of copper chelation therapy, which affects both tumor microenvironment and angiogenesis, may promote the efficacy of oncolytic virotherapy. In addition, serum copper levels have a detrimental effect on herpes virus infection. Based on these premises, it has been described that concomitant copper chelation therapy increases antitumor effect of herpes simplex virus–derived oncolytic viruses [151,152].

#### 2.5.5. Copper Depletion and Autophagy Inhibition

Autophagy has complex role in cancer development, progression and response to therapy. Autophagy inhibition is emerging as an effective approach for tumor therapy, particularly in cancers with increased levels of basal autophagy [153]. Different lines of evidence suggest that increased copper content activates a series of autophagy-related genes [154]. Accordingly, copper chelation using TM has been shown to inhibit the Unc-51-like autophagy activating kinase 1 and 2 (*Ulk1/2*) in lung adenocarcinoma cells [155]. Recently, the combination of copper chelation with TM and autophagy inhibition by chloroquine has been evaluated to promote pancreatic cancer cells death [156].

**Table 3:** Major preclinical studies on copper chelation therapy for cancer.

Tumor type	Drug/Intervention	Reference
Breast cancer	TM	[157]
*BRAF^V600E^* melanoma	TM	[121,158]
*BRAF^V600E^* papillary thyroid cancer	TM	[123]
*BRAF^V600E^* colon cancer	TM	[122]
Head and neck	TM	[159,160,161]
Endothelial and tumor cells	ATN-224	[162]
Lung cancer and head and neck carcinoma	TM + radiotherapy	[147,163]
Esophageal squamous cell carcinoma	TM + cisplatin	[164]
Gynecologic cancers	TM + cisplatin	[165]
Head and neck carcinoma	TM + OV	[151,152]
Head and neck carcinoma	TM + cetuximab	[148]
Colorectal cancer	Disulfiram + oxaliplatin	[166]
Hepatocellular carcinoma	TETA	[167]
Brain tumor	DPA	[168]
Mesothelioma	DPA, TETA or TM	[169]
Pancreatic duct adenocarcinoma	TM + CQ	[156]

Abbreviations: ATN-224: choline tetrathiomolybdate; CQ: chloroquine; DPA: D-penicillamine; OV: Oncolytic virotherapy; TETA: triethylenetetramine dihydrochloride, trientine; TM: Tetrathiomolybdate.

**Table 4:** Copper chelation therapy clinical trials for cancer.

Tumor Type	Trial Phase	Patients Enrolled	Drug/Intervention	Reference
Metastatic solid tumors including breast, colon, lung, and prostate cancers	I	18	TM	[131]
Renal cancer	II	15	TM	[170]
Breast cancer	II	75 + 40	TM	[113,171,172]
Prostate	II	19	TM	[173]
Mesothelioma	II	30	TM (poa)	[174]
Esophageal cancer	II	69	TM (poa)	[175]
BRAF melanoma	I	wd	Vemurafenib + TETA	NCT02068079
Metastatic colorectal cancer	I	24	TM + irinotecan, 5-FU, and IFL	[130]
Platinum-resistant epithelial ovarian cancer	I	5	TETA plus carboplatin	[137]
Head and neck, non-small cell lung and epithelial ovarian	I	55	TETA plus carboplatin	[138]
Relapse of epithelial ovarian, tubal, and peritoneal cancer	I	18	TETA plus carboplatin and PLD	[139]
Glioblastoma	II	40	DPA	[176]
Solid tumors including melanoma and breast, colon, kidney cancers	I	18	ATN-224	[177]
Relapsed prostate cancer	II	47	ATN-224	[178]

Abbreviations: 5-FU: 5-fluorouracil; ATN-224: choline tetrathiomolybdate; DPA: D-penicillamine; IFL: leucovorin; poa: post-operative administration; NCT number: ClinicalTrials.gov Identifier; PLD: pegylated liposomal doxorubicin; TETA: triethylenetetramine dihydrochloride, trientine; TM: tetrathiomolybdate.

## 3. Conclusions

Copper imbalance in Wilson’s disease has been well investigated, leading to the introduction of copper chelation therapy as a primary therapeutic tool which has significantly reduced morbidity, making Wilson’s disease a treatable disorder. Current efforts are focused on evaluating new chelating compounds and formulations to reduce toxic side effects, enhance ability to pass through the blood–brain barrier and improve patient’s compliance. A state of systemic or tissue-specific copper increase can occur through multiple mechanisms in addition to the genetic defects of copper metabolism observed in Wilson’s disease. Dysregulation of copper homeostasis has been observed in a wide spectrum of neurological, fibrotic pulmonary and vascular diseases as well as in different types of cancers. In these conditions, copper chelation should be ideally able to restore ionic balance by precise modulation of copper homeostasis. Unfortunately, the limited current knowledge of the complex mechanisms regulating neurodegenerative diseases, including Alzheimer’s and Parkinson’s diseases, and of the precise role or consequences of the mechanisms specifically dysregulated by copper imbalance in these brain pathologies have led to a minor success of the use of copper chelating agents for the treatment of these diseases. Similarly, the effect of deregulation of copper homeostasis in cancer seems to be multifaceted embracing tumor development, progression, angiogenesis, tumor microenvironment remodeling and metastasis. Copper chelating therapy has been proved to have antitumor effects mainly via disruption of angiogenesis and impaired migration, but further randomized clinical trials are necessary to confirm the benefit observed in preclinical models.

## Figures and Tables

**Table 1:** Main copper chelating drugs.

Compound Name	Abbreviation	Chemical Formula	Structural Formula
D-penicillamine: (S)-2-amino-3-mercapto-3-methylbutanoic acid	DPA	C_5_H_11_NO_2_S	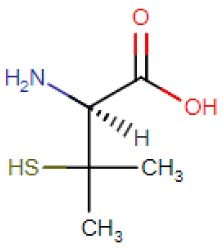
Tetrathiomolybdate	TM	MoS_4_	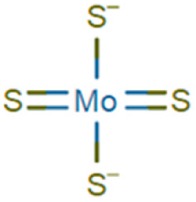
Trientine: triethylenetetramine dihydrochloride	TETA	C_6_H_18_N_4_	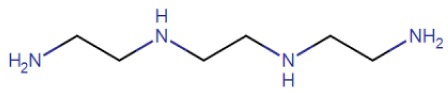
5,7-Dichloro-2[(dimethylamino)methyl]quinolin-8-ol	PBT2	C_12_H_12_Cl_2_N_2_O	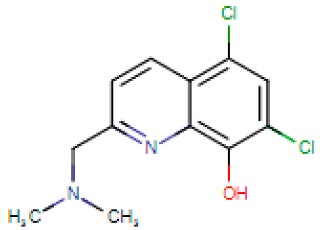
2,3-Dimercaptosuccinic acid	DMSA	C_4_H_6_O_4_S_2_	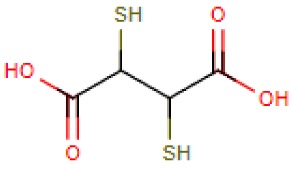

Structural formulas collected from the DrugBank public database (http://www.drugbank.ca/).

**Table 2:** Copper chelation therapy clinical trials for non-tumoral disorders.

Condition	NCT Number/Reference	Trial Phase	Patients Enrolled	Drug/Intervention	Status
Wilson’s Disease	NCT02273596	II	28	WTX101	completed
	NCT03299829	n.a.	50	TETA	recruiting
	NCT01472874	n.a.	8	TETA	completed
	NCT01378182	n.a.	10	MSC transplant	completed
Alzheimer’s disease	[60]	n.a.	34	DPA	terminated
	NCT00471211 [61]	n.a.	78	PBT2	completed
Idiopathic pulmonary fibrosis	NCT00189176	I/II	23	TM	completed
Diabetes Mellitus	NCT01295073	II	0	TETA	withdrawn
	NCT01213888	n.a.	5	TETA	terminated

Abbreviations: DPA: D-penicillamine; MSC: mesenchymal stem cells; n.a.: not available; NCT number: ClinicalTrials.gov Identifier; PBT2: 5,7-dichloro-2-[(dimethylamino)methyl]quinolin-8-ol; TETA: trientine tetrahydrochloride; TM: tetrathiomolybdate; WTX101: bis-choline tetrathiomolybdate.

## Abbreviations

AD	Alzheimer’s Disease
ATN-224	Choline tetrathiomolybdate
ATP7A/7B	Copper-transporting P-type ATPases 7A and 7B
BRAF	V-Raf Murine Sarcoma Viral Oncogene Homolog B1
Cp	Ceruloplasmin
Cu	Copper
CNS	Central Nervous System
CTR1	Copper transport protein 1
EMT	Epithelial mesenchymal transition
IPF	Idiopathic pulmonary fibrosis
DM	Diabetes mellitus
DPA	D-penicillamine
LOX	Lysyl Oxidase
LOXL	Lysyl Oxidase-Like
MAP2K1	Mitogen-activated protein kinase kinase 1
PD	Parkinson’s disease
ROS	Reactive oxygen species
SPARC	Secreted Protein Acidic and Rich in Cysteine
TETA	Triethylenetetramine
